# Elevated Percentage of CD3^+^ T-Cells and CD4^+^/CD8^+^ Ratios in Multiple System Atrophy Patients

**DOI:** 10.3389/fneur.2020.00658

**Published:** 2020-07-07

**Authors:** Bei Cao, Xueping Chen, Lingyu Zhang, Qianqian Wei, Hui Liu, Weihua Feng, Yongping Chen, Huifang Shang

**Affiliations:** ^1^Department of Neurology, West China Hospital, Sichuan University, Chengdu, China; ^2^Department of Laboratory Medicine, West China Hospital, Sichuan University, Chengdu, China

**Keywords:** multiple system atrophy, humoral immune, cellular immune, prevalence, disease process

## Abstract

α-synuclein is involved in the pathogenesis of multiple system atrophy (MSA) and can be regulated by peripheral immune activation (PIA). We aimed to clarify the correlations between PIA and the prevalence of MSA and to analyze the role of PIA in the progression of the disease. A total of 321 patients with probable MSA and 321 age- and gender-matched healthy controls were included in this study. Lymphocyte subsets, including CD3^+^, CD4^+^, and CD8^+^ cells, and the levels of immunoglobulins IgG, IgM, and IgA were evaluated. The proportions of CD3^+^ and CD4^+^ T-lymphocytes were significantly increased in MSA patients compared with those of normal controls. In addition, the ratio of CD4^+^ to CD8^+^ cells was significantly increased in male MSA patients and IgG concentrations were decreased in female MSA patients. Furthermore, the concentrations of IgM in female MSA patients were dynamically different at various disease stages and gradually decreased from the early stage until the end stage of the disease (*p* = 0.029). Other detected immunological indexes were not significantly different during the entire disease course. In this study, high proportions of CD3^+^ and CD4^+^ T-lymphocytes and decreased IgG levels were associated with an increased risk for MSA in a Chinese patient population. In addition, PIA may be involved in the progression of MSA.

## Introduction

Multiple system atrophy (MSA) is a late-onset, sporadic neurodegenerative disease that manifests as autonomic failure and a variable presence of poorly levodopa-responsive parkinsonism and/or cerebellar ataxia. Neuropathologically, MSA is defined by striatonigral and/or olivopontocerebellar neurodegeneration and widespread and abundant α-synuclein-positive cytoplasmic inclusions in the glia cells of the central nervous system (CNS) (1). The two clinical subtypes of MSA, the parkinsonian variant (MSA-P) and the cerebellar variant (MSA-C), are distinguished by their predominant motor features. These variants, along with Parkinson's disease (PD) and dementia with Lewy bodies (DLB), are also referred to as synucleinopathies (2).

The pathologic mechanisms of synucleinopathies are largely unknown but chronic neuroinflammation is likely involved in these diseases (3). Therapeutically, inflammasome inhibition prevents α-synuclein pathology and dopaminergic neurodegeneration in an animal model (4) and combined active humoral and cellular immunization approaches for the treatment of synucleinopathies by reducing the accumulation of α-synuclein has shown some potential (5), suggesting a key role of neuroinflammation in these diseases.

Till now, in the CNS, immune and inflammatory responses involved in MSA has been intensively investigated. For example, an altered expression of multiple Toll-like receptors (TLRs) has been reported in brain sections from MSA patients, including the substantia nigra (SN), the striatum, the cerebral cortex, and the nucleus dentatus (6). Previous studies found that inflammation-related genes are up-regulated in the rostral pons which undergoes extensive damage in MSA (7). In addition, dysregulated expression of genes associated with neuroinflammation in SN and striatum was found even in non-symptomatic disease stage in an MSA mouse model (8). Very recently, a significant increase of HLA-DR^+^ microglia, CD3^+^, CD4^+^, and CD8^+^ T cells in the putamen and SN of MSA patient tissue compared to controls were reported (9), indicated adaptive immunity involved in the pathogenesis. However, systematically investigating the role of peripheral immune in MSA remains insufficient.

As we all know, blood-brain barrier (BBB) protects neurons from factors present in the systemic circulation, and maintains the highly regulated CNS internal milieu. Pathologically, as shown in PD and other neurodegenerative diseases, multiple molecular pathways induced by BBB disruption, allows influx into the brain of neurotoxic blood-derived debris, cells and microbial pathogens and is associated with inflammatory and immune responses, which can initiate neurodegeneration (10). Conversely, pathological products, such as α-synuclein, could easily enter into the blood from brain (11) and activate peripheral immune (12), which might further aggravate deterioration of systemic conditions. In addition, dysautonomia in MSA could have contributed to immune dysregulation, given the effect of sympathetic and parasympathetic innervation of major lymphoid organs. As accumulated evidences suggested that peripheral immunity is involved in the pathogenesis of PD (13), it was reasonable to suppose abnormal peripheral immune activation (PIA) contribute to the development for MSA. Therefore, in this study we will devote to explore: (1) to compare whether the proportion of T-cell subsets and the levels of serum immunoglobulins IgG, IgM, and IgA in MSA patients and normal controls are different, and (2) to test whether the PIA state of MSA patients would correlate with the disease subtype as well as to analyze the role of PIA in the progression of the disease.

## Methods

### Patients and Healthy Individuals

This study was performed within the Department of Neurology, West China Hospital of Sichuan University, which is a comprehensive medical teaching and research center and a leading medical center of West China. A total of 321 probable MSA patients were recruited from May 2009 to November 2016, all of them met the current consensus criteria for MSA by Gilman et al. (14), which comprise exclusion criteria (including prominent slowing of vertical saccades, vertical supranuclear gaze palsy, and evidence of focal cortical dysfunction) to increase the specificity of the diagnosis of MSA. All the patients were then followed up by using telephone or face-to-face interviews during a 1-year interval by our neurologists until 31st December 2018. A total of 321 age- and gender-matched healthy controls without any neurological conditions were recruited from the medical examination center of West China Hospital. All patients and control subjects gave their written informed consent. The study protocol was approved by the Ethical Committee of West China Hospital of Sichuan University (approval no. 2015-236). The datasets analyzed during the current study are available from the corresponding author on reasonable request.

### Exclusion Criteria

Patients who showed evidence of systemic inflammation during a clinical examination or blood biochemical tests (such as an increased number of white blood cells, a high concentration of C-reactive protein, or a high erythrocyte sedimentation rate) were excluded. Patients with manifestations of monoclonal gammopathy, non-malignant endocrine abnormalities, neoplastic disorders, auto-antibodies (high-titer of GM1 ganglioside antibody), or infection (HIV-1, hepatitis B and C, varicella zoster, syphilis, or borreliosis) were also excluded. None of the patients had a history of using anti-inflammatory drugs, steroids, acetylsalicylic acid, or statins during the last 2 months before enrollment.

### Collection and Processing of Blood Specimens

In accordance with the study protocols, peripheral blood samples (~9 mL each set) were obtained by venipuncture performed between 9:00 and 11:00 a.m. after fasting from midnight from each patient and healthy control participant. Blood was collected into a sterile 7.5 mL tube with a clot activator and double gel for transport (Becton Dickinson (BD) Vacutainer, SST, REF 367987). All experiments in this study were conducted in the Division of Clinical Immunology, West China Hospital, affiliated to Sichuan University. Cell viability was analyzed via flow cytometric assays. The lymphocyte subsets were identified and determined using the BD FACSCanto™ II Flow Cytometer (BD Biosciences, Heidelberg, Germany). For the flow cytometry analyses, the reagent cocktail (10 μL) containing CD4-fluorescein isothiocyanate (FITC), CD8-phycoerythrin (PE), and CD3 peridinin chlorophyll protein (PerCP) was added to 50 μL heparin anticoagulated whole blood. The lymphocytes were gated as forward-scattered light and side-scattered light. For each sample, at least 10,000 cells were analyzed and the percentage of the cells expressing CD3^+^, CD4^+^, and CD8^+^ markers were evaluated. The levels of immunoglobulins IgG, IgM, and IgA were quantified via nephelometry on an Image 800 nephelometer (Beckman Coulter, Fullerton, California). A portion of each collected sample was analyzed to detect infectious diseases. According to the manufacturer's instructions, all tests were performed by board-certified laboratory technicians who were blinded to the clinical data.

### Clinical Evaluation

The relevant demographic features and clinical data were collected, including age, sex, age of onset, and disease duration. Disease duration was defined as the time from symptom onset to the baseline evaluation in the present study. Survival time was defined as the time from the date of onset to the date of death. Based on their clinical symptoms, MSA patients were classified into two subgroups: MSA-C, characterized predominantly by cerebellar ataxia, and MSA-P, characterized predominantly by parkinsonism. Disease severity was evaluated by a neurologist using the united multiple system atrophy rating scale (UMSARS; subscale I = activities of daily living, II = motor section, and IV = disability).

### Statistical Analysis

The distribution of the detected variables of PIA, including the T-lymphocyte subset frequencies as well as IgG, IgM, and IgA levels, were tested for normality using Shapiro–Wilk tests. Grubbs' test was used to determine if a minimum value or a maximum value was an outlier. Student's *T*-test was applied to analyze the differences in the detected variables of PIA between MSA patients and healthy controls if the variables were normally distributed. Chi-square tests were used to compare sex ratios between MSA and control groups. One-way ANOVA was performed to compare each detected PIA variable among MSA patients with different stages. Spearman correlation analysis was used to assess the relationship between lymphocytes/immunoglobulin levels and clinical features. The Bonferroni method was used to correct for multiple comparisons by adjusting the significance level of multiple comparisons (Pc = P × n, *n* was the number of tested comparisons). All data is presented in the form of mean ± standard deviation and were analyzed using SPSS 23.0. A *p*-value of <0.05 (for multiple comparisons, the Pc should be <0.05) as considered to be statistically significant.

## Results

The demographic features of the MSA patients and the healthy controls are listed in Table 1. The mean disease duration and UMSARS scores of MSA patients obtained when blood sampling were 2.5 ± 1.0 years and 37 ± 27, respectively. No significant differences were observed in age and sex between MSA patients and the control subjects (Table 1).

**Table 1:** Demographic characteristics and PIA variables of patients with MSA and control subjects.

	**MSA patients (*****n*** **=** **321)**			**Controls (*****n*** **=** **321)**			P-value^*^		
	**All subjects**	**Men (*n* = 162)**	**Women (*n* = 159)**	**All subjects**	**Men (*n* = 162)**	**Women (*n* = 159)**	***P*_**ALL**_**	***P*_**M**_**	***P*_**W**_**
Age (years)	58.9 ± 8.5	58.9 ± 9.0	58.9 ± 7.0		58.6 ± 7.7	58.0 ± 7.0	0.340		
% CD3	65.3 ± 10.0	65.1 ± 9.6	65.4 ± 10.4	61.9 ± 9.9	60.9 ± 10.3	62.9 ± 9.3	<0.0001	<0.0001	0.008^#^
% CD4	37.0 ± 8.7	36.9 ± 8.6	37.3 ± 8.8	32.2 ± 7.0	31.4 ± 7.1	33.0 ± 7.0	<0.0001	<0.0001	0.001
% CD8	23.9 ± 8.2	24.0 ± 8.5	23.7 ± 7.8	24.2 ± 7.0	24.5 ± 8.0	24.0 ± 7.7	0.311	0.280	0.744
CD4/CD8	1.8 ± 0.9	1.8 ± 0.9	1.7 ± 0.9	1.5 ± 0.6	1.4 ± 0.5	1.5 ± 0.6	<0.0001	0.002	0.060
IgG (g/L)	11.9 ± 2.6	11.8 ± 2.7	11.9 ± 2.5	12.8 ± 2.3	12.3 ± 2.3	13.3 ± 2.2	<0.0001	0.049^#^	0.001
IgA (mg/L)	2206.9 ± 1249.3	2176.7 ± 1376.5	2237.8 ± 1108.3	2249.2 ± 1083.0	2059.7 ± 737.8	2442.3 ± 1321.5	0.244	0.536	0.310
IgM (mg/L)	1258.0 ± 650.2	1189.9 ± 557.8	1333.2 ± 728.6	1291.9 ± 576.0	1196.1 ± 459.2	1389.4 ± 661.9	0.366	0.562	0.453
Age at onset	56.1 ± 8.4	56.2 ± 9.0	56.1 ± 7.9						
MSA-C/MSA-P	196/125	109/53	87/72						
Disease duration (years)	2.5 (1.0)	2.6 (1.7)	2.5 (2.3)						
UMSARS	37 (21)	36 (16.5)	40 (24)						
Survival time (years)	5.7 ± 1.4	5.8 ± 1.3	5.7 ± 1.2						

MSA, multiple system atrophy; P_ALL_, all subjects' comparison between MSA patients and Controls; P_M_, men comparison between MSA patients and Controls; P_W_, women comparison between MSA patients and Controls; PIA, peripheral immune activation;

* the significant level was corrected with the formula of α′ = α × 21 (21 tests in the table, 7 parameters × 3 tests/parameter) according to the Bonferroni method, when α′ was <0.05 was considered as statistically significant;

# *no statistical significance after Bonferroni correction*.

The results for the detected variables of PIA in MSA patients and controls are summarized in Table 1. Analysis of T-cell subsets showed that MSA patients had significantly higher proportional levels of CD3^+^ and CD4^+^ T-lymphocyte subsets when compared to healthy controls (*p* < 0.0001 and *p* < 0.0001, respectively). These findings were mainly seen in male patients. The percentages of CD8^+^ cytotoxic T-cells were not different either between MSA patients and controls or between their subtypes evaluated separately based on gender. Consequently, the CD4^+^/CD8^+^ ratio was significantly higher in the MSA group (*p* < 0.0001). In gender-specific analyses, a significant elevation of the CD4^+^/CD8^+^ ratio was observed in male MSA patients (*p* = 0.002) but not in female MSA patients. IgG concentrations were significantly lower in MSA patients than in the control subjects (*p* < 0.0001). In particular, a significant decrease in IgG concentration was observed in females (*p* = 0.001) but not in male patients. There were no significant differences in IgA and IgM concentrations between MSA patients and control subjects.

In the subtype analysis, the UMSARS scores of patients with MSA-P were significantly higher than those of patients with MSA-C (*p* < 0.0001) were observed, indicating a more severe disease status in MSA-P than in MSA-C patients. Furthermore, significant differences, including elevated percentages of CD3^+^ and CD4^+^ T-cells, increased CD4^+^/CD8^+^ ratios and decreased concentrations of IgG, were identified in MSA-C and MSA-P patients compared to healthy control subjects. However, no differences were found between MSA-C and MSA-P patients (**data not shown**).

**Figure 1:**
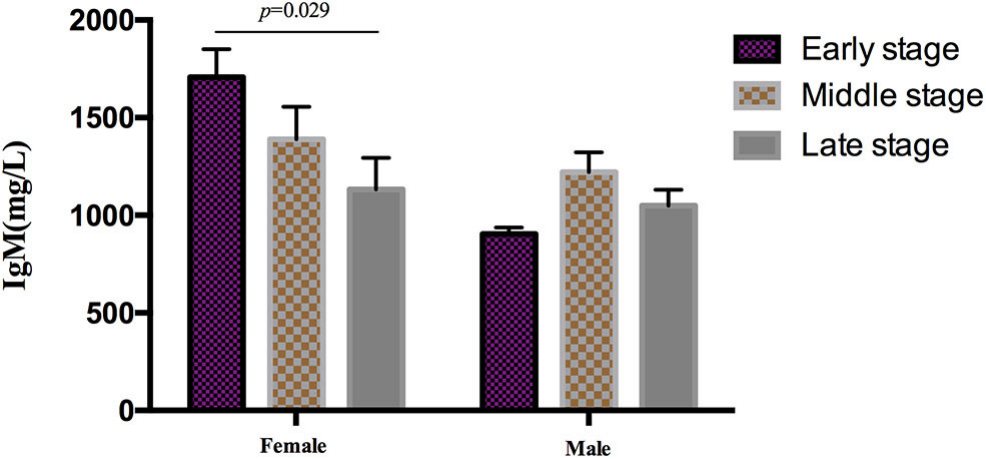
Differential expression of IgM in different clinical stages MSA patients according to sex. IgM levels gradually decreased from early to later stages exclusively in female (*p* = 0.029), no significant difference was found in male patients.

During the analysis of the PIA data, 119 patients died. The mean survival time of MSA patients was 5.7 ± 1.4 years. To date, there is no recognized disease staging system for MSA. To clarify the role of peripheral immune factors in the development and progression of MSA, we artificially defined disease duration as early (0 < *x* < 1/3), middle (1/3 ≤ *x* ≤ 2/3), or late stage (2/3 < *x* < 1) when the patients were sampled for testing the immunological indexes (x is defined as the ratio of disease duration to survival time). According to this method, the mean age, the mean disease duration, and the mean UMSARS of patients were gradually increased from early to late stages and significant differences were found in the mean disease duration and the mean UMSARS of patients at different stages (Table 2), providing support to this clinical staging method. We also found that IgM levels gradually decreased from early to late stages (*p* = 0.026). We also analyzed the PIA variables of MSA patients according to sex and found this significant difference of IgM exclusively in female patients (*p* = 0.029, Figure 1). In addition, there was no significant difference in immune indexes expression between MSA-C and MSA-P patients (Table 2).

**Table 2:** Demographic characteristics and PIA variables of different stages and subtypes in dead MSA patients.

**Variable**	**Disease stages**							**Subtypes**		***P***
	**Early stage**	**Middle stage**	**Later stage**	**DFE**	**DFA**	**F**	***p***	**MSA-P**	**MSA-C**	
Age	57.7 ± 6.4	59.3 ± 8.3	61.2 ± 7.5	117	2	1.334	0.267	61.0 ± 7.7	58.7 ± 7.8	0.111
Age at onset	56.2 ± 6.4	56.3 ± 8.1	56.4 ± 7.4	117	2	0.004	0.996	57.5 ± 7.7	55.3 ± 7.5	0.124
Male/Female	3/12	30/34	24/16				0.029^*^	23/32	34/30	0.218
MSA-P/MSA-C	8/7	28/36	19/21				0.783	−	−	
Disease duration	1.5 ± 0.5	3.0 ± 0.9	4.8 ± 1.1	117	2	90.289	<0.0001	3.5 ± 1.4	3.4 ± 1.4	0.621
UMSARS	40.2 ± 10.0	46.3 ± 15.4	57.0 ± 18.3	117	2	8.237	<0.0001	53.3 ± 17.7	45.5 ± 17.1	<0.0001
% CD3	63.9 ± 7.8	66.4 ± 10.2	62.4 ± 10.0	117	2	1.977	0.164	63.9 ± 10.2	65.4 ± 9.8	0.410
% CD4	38.7 ± 8.5	36.6 ± 9.4	35.8 ± 8.3	117	2	0.580	0.522	37.3 ± 9.3	35.8 ± 8.5	0.362
% CD8	21.7 ± 7.7	25.1 ± 9.4	22.8 ± 7.5	117	2	1.405	0.311	22.5 ± 7.2	25.1 ± 9.2	0.110
CD4/CD8	2.0 ± 0.9	1.7 ± 1.0	1.7 ± 0.8	117	2	0.805	0.325	1.9 ± 1.0	1.7 ± 0.9	0.205
IgG (g/L)	10.8 ± 2.1	11.8 ± 2.6	12.1 ± 3.0	117	2	1.268	0.262	11.7 ± 2.6	11.9 ± 2.8	0.618
IgA (g/L)	1906.0 ± 630.3	2150.3 ± 1259.7	2534.6 ± 1551.9	117	2	0.198	0.344	2161.1 ± 1177.0	2324.0 ± 1433.8	0.504
IgM (g/L)	1547.2 ± 571.6	1313.6 ± 782.0	1072.1 ± 509.4	117	2	3.138	0.026	1235.6 ± 666.4	1276.9 ± 707.1	0.745

*PIA, peripheral immune activation; DFE, degrees of freedom for error; DFA, degrees of freedom for analysis*.

* *indicates no significanct difference after Bonferroin correction*.

To better understand the relationship between PIA and MSA, we assessed correlations between lymphocytes/immunoglobulin levels and clinical features, including disease duration, age at onset, disease severity, and disease duration to death. Except for a negative correlation found between CD4^+^ and onset age (*R* = −0.151, *p* = 0.007), there were no other correlations between these immune indexes and disease duration, disease duration to death, or disease severity (Table 3).

**Table 3:** Spearman correlations of clinical characteristics with PIA variables of MSA patients.

**Variable**	**Age at onset**		**Disease duration**		**UMSARS**		**Survival time**	
	**R**	***p***	**R**	***p***	**R**	***p***	**R**	***p***
% CD3	−0.077	0.174	−0.022	0.700	−0.021	0.711	0.111	0.235
% CD4	−0.151	0.007	−0.018	0.755	−0.032	0.571	−0.023	0.809
% CD8	0.097	0.084	−0.061	0.277	0.001	0.981	0.066	0.478
CD4/CD8	−0.135	0.015	−0.004	0.949	−0.042	0.454	−0.038	0.685
IgG (g/L)	0.108	0.054	0.0001	0.955	−0.054	0.336	−0.003	0.975
IgA (g/L)	0.076	0.175	0.066	0.237	0.038	0.496	0.0001	0.999
IgM (g/L)	−0.099	0.076	−0.099	0.076	−0.034	0.547	−0.052	0.575

## Discussion

To our knowledge, the current study included the largest datasets to determine whether peripheral immune abnormalities are involved in MSA through the evaluation of T-cell subsets and assessment of humoral immunity in MSA patients.

In this study, we found an increase in CD3^+^ and CD4^+^ T-lymphocyte population levels, no change in CD8^+^ T-cytotoxic cell subpopulation levels, and consequently an increase in the CD4^+^/CD8^+^ ratio in our MSA patients, indicating an enhanced immune activation in the peripheral system (15, 16). To date, there is no direct evidence for the involvement of T-lymphocyte subsets in the CNS in the pathogenesis of MSA. Previously, researchers have observed peripheral leukocyte migration into the brain and CD4^+^/CD8^+^ T-cell infiltration of the SN in PD patients and mouse models (17–19). In neurodegenerative diseases, inflammatory and immune responses mediated by the breakdown of the BBB can initiate multiple pathways of neurodegeneration (10). Therefore, similar to PD, enhanced peripheral immunity may also involve in the pathogenesis of MSA. However, the mechanisms by which activated T-lymphocytes regulate the disease remain unknown. Increased levels of major histocompatibility complex class II positive cells, including macrophages and microglia cells in the dorsomedial prefrontal cortex of MSA patients, can disrupt the BBB and attract CD4^+^ T-lymphocytes to the site of neuronal injury (20), which may lead to decreased CD3^+^ or CD4^+^ levels in the periphery. Alternatively, the drainage of aberrant forms of α-synuclein found in MSA patients from the CNS into the lymphatic system could allow the protein to activate lymphocytes to mobilize the adaptive immune system. Based on the theory of Braak's staging in PD or AD, where the pathogenesis develops from the peripheral system to the CNS (21), PIA has been suggested to play a role in the etiology of MSA. This could explain the enhanced adaptive immunity even in the very early stages of the disease, such as increased percentages of CD3^+^ and CD4^+^ T-lymphocytes and elevated CD4^+^/CD8^+^ ratios. With regards to other neurodegenerative diseases, circulating lymphocytes have also been found to be decreased in PD patients, mainly of the “naive” helper T-cell phenotype (CD4^+^CD45RA^+^) (22). However, either increase or decrease of T-lymphocyte subsets could be explained by the aforementioned theories. Further studies are needed to determine if there is an increase or decrease in T-lymphocyte subsets in the periphery in different stages of MSA, which might specify the underlying mechanisms of the disease. Although the proportion of T-lymphocyte subsets in different disease stages was analyzed in this study, there was no significant difference due to the limited sample size.

In addition, a significant decrease in IgG concentrations was found in MSA patients, especially in female patients, reflecting different immunological profiles concurrent with predominant humoral or cellular immune responses compared with healthy subjects. Although sex differences in immune responses remain incompletely understood, sexual dimorphism in immune response and host susceptibility may be caused by a sex-specific genetic architecture. It is known that the X-chromosome encodes more than 1,000 genes related to immunity and one more X-chromosome may provide the added biological advantage observed in female MSA patients (10). As our results show, there is a higher expression of immune factors in healthy females than in males (23). Therefore, gender-specific alterations of serum antibodies may account for the increased prevalence of autoimmune diseases in women but further research is required to gain a detailed understanding of the influence of gender in systemic humoral immune responses in MSA. In addition, previous studies found improved UMSARS in MSA patients after administration of intravenous immunoglobulin, which indicates that IgG may also play a role in α-synuclein clearance and neuronal protection in MSA (24). Thus, decreased IgG concentrations in serum may disturb the fine balance between the beneficial and detrimental aspects of the immune response in the α-synucleinopathy that occurs in MSA. Therefore, our results would suggest that humoral immunity may be impaired in MSA patients.

In this study, we identified the differential expression of peripheral immune indicators during the progression of MSA using a special clinical staging method. Interestingly, these immune factors, including CD3^+^, CD4^+^, and IgG, were significantly different between MSA patients and healthy controls but did not change between different disease stages, suggesting that these factors could be useful indicators for diagnosis of MSA during the entire disease progression. However, as we have shown in this study that a correlation exists between CD4^+^ and onset age, the variant baseline value of T-lymphocyte subsets should be considered in different patients. In addition, these results should be tested in other autoimmune diseases or neurodegenerative diseases and additional biomarkers should also be investigated to improve the diagnostic efficiency for MSA. Furthermore, we found that IgM levels gradually decreased from the early stage to the late stage in the disease process, indicating that the immune responses of MSA mainly occurred at early stage since IgM represents the acute phasic protein for the inflammatory response. This finding would therefore support the recommendation of immunotherapy for MSA at the early stage of the disease.

Some limitations of our study should be considered. First, our “artificial” staging method should be validated in further studies, such as cross-validation. Therefore, the results regarding the longitudinal effects of the peripheral immune system also need to be confirmed by using other clinical staging methods or by longitudinal sampling in different stages in the same patients. Secondly, other factors that may influence the peripheral immunity, such as smoking, drug administration (e.g., L-dopa, dopaminergic receptors agonist, psychotropic medications, antibiotics exposure) were not adjusted for in the statistical analysis. Finally, the abnormal peripheral immunity observed need to be more studies which explain the potential neuropathogenic mechanisms.

## Conclusions

MSA patients had higher levels of CD3^+^ and CD4^+^ T-lymphocytes, increased CD4^+^/CD8^+^ ratios, and lower IgG levels than healthy controls, suggesting the involvement of the peripheral immune system in the pathogenesis of MSA. In addition, dynamic alterations in IgM levels indicated that humoral immunity may be involved in the progression of MSA. Further prospective studies with larger sample sizes should be conducted to confirm our findings.

## Data Availability Statement

The datasets generated for this study are available on request to the corresponding author.

## Ethics Statement

The studies involving human participants were reviewed and approved by Ethical Committee of West China Hospital of Sichuan University. The patients/participants provided their written informed consent to participate in this study.

## Author Contributions

HS and BC designed the study. BC did the statistics and wrote the manuscript and HS revised the manuscript. XC, LZ, QW, HL, and WF collected the data. YC analyzed the data, reviewed, and critiqued the manuscript. All authors contributed to the article and approved the submitted version.

## Conflict of Interest

The authors declare that the research was conducted in the absence of any commercial or financial relationships that could be construed as a potential conflict of interest.
